# Enhancing accuracy and convenience of golf swing tracking with a wrist-worn single inertial sensor

**DOI:** 10.1038/s41598-024-59949-w

**Published:** 2024-04-22

**Authors:** Myeongsub Kim, Sukyung Park

**Affiliations:** Department of Mechanical Engineering, Korea Advanced Institute of Science and Technology, Daejeon, 34141 South Korea

**Keywords:** Mechanical engineering, Health care

## Abstract

In this study, we address two technical challenges to enhance golf swing trajectory accuracy using a wrist-worn inertial sensor: orientation estimation and drift error mitigation. We extrapolated consistent sensor orientation from specific address-phase signal segments and trained the estimation with a convolutional neural network. We then mitigated drift error by applying a constraint on wrist speed at the address, backswing top, and finish, and ensuring that the wrist's finish displacement aligns with a virtual circle on the 3D swing plane. To verify the proposed methods, we gathered data from twenty male right-handed golfers, including professionals and amateurs, using a driver and a 7-iron. The orientation estimation error was about 60% of the baseline, comparable to studies requiring additional sensor information or calibration poses. The drift error was halved and the single-inertial-sensor tracking performance across all swing phases was about 17 cm, on par with multimodal approaches. This study introduces a novel signal processing method for tracking rapid, wide-ranging motions, such as a golf swing, while maintaining user convenience. Our results could impact the burgeoning field of daily motion monitoring for health care, especially with the increasing prevalence of wearable devices like smartwatches.

## Introduction

Golf is a sport that is greatly influenced by the swing mechanics of multi-body segments and the club. Players and coaching staffs have a high demand for golf swing monitoring to improve golf performance and prevent injuries^1^. In recent years, with the development of micro sensors and smart devices, there has been an exponential surge in the demand for sophisticated swing monitoring technologies. This has led to a rapid increase in the development of golf monitoring products and golf biomechanics researches^1–11^. Advancements in image processing and deep learning technologies have recently led to numerous studies on methods of golf swing monitoring, such as joint trajectories and swing postures, and club trajectories, etc. Jia et al. estimated the wrist trajectory during a golf swing to be around 17 cm and 10 cm error levels, respectively, through the use of a depth camera alone and in combination with inertial sensors^11^. Park et al. applied a machine learning algorithm, random forest, to depth camera images to estimate the positions of key joints during a golf swing, with an error level of approximately 3 cm^12^. Ko et al. estimated the rotation angles of multiple body segments from images of a single RGB camera, with an error level of 3–8° per axis^13^. Nam et al. estimated the club trajectory to an error level of 13.2 cm by integrating information from an inertial sensor attached to the club and stereo camera images^14^. Although the development of artificial intelligence technologies has led to an increase in research on image-based golf tracking, the use of sensors such as cameras to obtain image data limits the convenience of monitoring golf swings in the field. To ensure the convenience of golf swing monitoring, wearable devices based on inertial sensors are being utilized in the field of golf swing monitoring. Kim et al. analyzed the accelerations during golf swing movements using two inertial sensors attached to the left and right wrists, and calculated the average swing trajectory^15^. King et al. calculated the putter head trajectory using an inertial sensor located at the end of the club during robotic putting^16^. Jensen et al. estimated the time spent in each phase of putting using machine learning algorithm applied to the inertial sensor data acquired from the putter head^17^. Cheon et al. estimated the club head trajectory using a single inertial sensor attached to the grip part of the club^18^. Jia et al. examined technical issues during an inertial sensor-based golf swing, reporting that the accuracy of trajectory estimation is decreased due to noise from the inertial sensor and that a 30–60 cm level of error occurs depending on the attachment location of the inertial sensor, given the large variability and fast action of golf swings^11^. Most of the previous studies have the location of the inertial sensor attachment on the club rather than the body and the information of the swing being monitored is mainly the acceleration or angular speed measured directly from the inertial measurement unit (IMU). Although trajectories of body segments during swing are more intuitive tracking information for golfers than velocity or acceleration, there are not many studies or commercial products that provide quantitative swing trajectory information, due to the limited accuracy in trajectory estimation.

In the pursuit of enhancing the accuracy of swing trajectory estimation using inertial sensors, particularly when the number and placement of sensors are constrained for user convenience, the following technical issues need to be addressed to improve accuracy: First, from the swing data measured by the inertial sensor, we need to robustly identify major sequences of a golf swing, such as the address (ADD), backswing top (BST), impact (IMP), and finish (FIN), despite differences in swing style according to skill level and type of club. Previous studies have attempted to perform segmentation using traditional signal processing methods, heuristic methods, and machine learning algorithms. Jensen et al. applied machine learning techniques to the inertial sensor data attached to the putter head to calculate the major phases of putting^17^, while Kooyman et al. used a heuristic method based on the zero-crossing moments of angular velocity^19^. Heuristic methods utilizing IMU signal threshold values have been applied to the segmentation of inertial sensor data attached to the club^20^ and the grip^20^. Other studies have explored segmentation of golf swing phases using IMUs attached to the body rather than the club. For instance, Kim et al. compared the accuracy of heuristic method, Convolutional Neural Network (CNN), and Bidirectional Long Short-Term Memory (BLSTM) in segmenting inertial sensor data from various body parts such as the wrist, waist, and head. They reported that machine learning-based segmentation demonstrated high accuracy^21^.

The second technical issue that needs to be addressed to improve accuracy is calibrating the sensor orientation. To calculate the swing trajectory from an inertial sensor attached to the body or club, it is necessary to know the sensor orientation that transforms the relative coordinate system at the attachment point to the absolute coordinate system of the ground. For this, the sensor orientation at the start of the motion, mostly the ADD, is first estimated, and then, using this value as an initial condition, the rotation of the sensor coordinate system over the entire range of the motion is calculated by integrating the angular velocity signal measured from the inertial sensor^22^. During the integration process, errors caused by bias and scale factors of the inertial sensor signals accumulate over time^23^, and there have been reports indicating that fast rotational motions with a large angular velocity result in more significant orientation errors^24^. The typical method of estimating the initial posture's sensor orientation involves temporarily placing the inertial sensor on a known coordinate system like the ground or a table, where only gravitational acceleration is present, for a few seconds^25^. Similarly, for estimating the orientation of an inertial sensor attached to the body, pose calibration methods, including the N-pose (standing posture with arms stretched vertically downwards) and the T-pose (standing posture with arms extended horizontally perpendicular to the body), are widely used before motion measurement^26,27^. There have also been suggestions to calibrate the orientation estimation through postural constraints during movements^28,29^. However, pose calibration can sometimes cause the estimation accuracy to depend on the user's body parameters^27^, and often interferes with a natural golf swing. Several studies have reported developing and applying filters for the estimation of orientation through the fusion of acceleration, angular velocity, and geomagnetic signals. Kalman filter-based methods have been applied to estimate the orientation of sensors attached to lower limbs and waist during walking^30–32^. In addition, Mahony filter^33^, and Madgwick filter^34^ have been suggested and are widely used. However, the corrective effect of these filter on golf swing movements, which are characterized by large acceleration changes over short periods, is limited^27^ due to the need for periodicity and convergence conditions for filter implementation^35^. Recently, studies have reported the application of machine learning techniques to extract feature points for motion recognition in various activities, including walking^36^, running^37^, golf^38^, tennis^39^, volleyball^40^, and cross-country^41^. Zimmermann et al. used CNNs and Long Short-Term Memory (LSTM) to estimate the alignment between IMUs and body segments during walking^42^. Though not a case of estimating sensor orientation in the ground-fixed coordinate, it is noteworthy because it demonstrates the possibility of extracting information about sensor direction from inertial sensor signals using convolutional neural networks.

The third issue to enhance the tracking accuracy is to correct the inherent drift error in swing trajectory estimates. The causes of drift errors that occur in the integration process of acceleration and angular velocity from inertial sensors include the accumulation of sensor noise such as bias, sensor alignment errors, among others, which increase proportionally with time. Specifically, golf swings, with their fast motion and wide 3D rotation, have been found to produce substantial trajectory tracking errors, often in the range of 30 to 60 centimeters^11^. In previous research monitoring walking or running activities based on inertial sensors, drift errors were corrected using the features of motion such as ground contact and periodicity. For instance, during walking, the foot's contact with the ground creates a recurring kinematic feature where vertical speed and position become zero. The Zero Velocity Update (ZUPT) method, which periodically updates the speed to zero at the point of ground contact, is widely used^30,43^. Abdulrahim et al. corrected drift errors by applying a Kalman filter based on the gravitational acceleration information during the stance phase^44^. Kose et al. adjusted the drift error in stride calculation by using the repeated speed at each gait cycle^31^. However, golf swings, unlike walking or running, lack repetitive actions such as ground contact, necessitating the development of novel methods to correct integration errors. Many studies on golf swing mechanics have identified recurring kinematic features among golfers, including the coordination of multi-joint motion and sequential rotations of body segments during swings^7,8,45–51^. It is worth to examine whether the observed swing kinematics could be used to mitigate drift errors in swing trajectories.

In this study, we addressed two technical issues associated with enhancing the accuracy of golf swing trajectories estimated using a wrist-worn IMU: orientation estimation and drift error reduction. To achieve this, we exploited the kinematic characteristics of golf swing incorporating with machine learning. Specifically, we proposed a method to estimate the sensor's orientation by extracting information of coordinate rotation embedded in the IMU data during a natural swing, without the need for the golfer to perform any pre-calibration actions such as N-pose or T-pose. We then proposed a drift reduction method by applying the kinematic characteristics of the upper limb during a golf swing. Finally, we quantitatively compared the accuracy of the swing's wrist trajectories as estimated by a single inertial sensor, applying both proposed techniques.

## Methods

We proposed solutions to two technical issues necessary for enhancing the accuracy of wrist trajectory tracking during a golf swing using a wrist-worn inertial sensor. The first step in calculating the wrist trajectory involves the segmentation of the main golf swing events from the sensor signal. This step should be followed by the conversion of the relative coordinate frame of the wrist-worn sensor into the ground-fixed absolute coordinate frame. Finally, the removal of the drift error from the wrist trajectory, which results from the integration of the acceleration signal, should be conducted. The two solutions proposed and verified herein are: firstly, to estimate the sensor orientation that transform the relative coordinates into absolute ones without the need for additional sensors or calibration actions; and secondly, to reduce the drift errors arising from the integration of the sensor data.

## Experiment

Twenty male right-handed golfers, with an average age of 41.2 ± 9.1 years, height 175.4 ± 6.9 cm, and weight 79.9 ± 13.4 kg, participated in data collection. To test the effectiveness of our proposed trajectory correction method with different swing patterns, we collected data from both professional and amateur golfers using a driver and a 7-iron. Nine professional golfers were members of the Korea Golf Association, and eleven amateur golfers had an average handicap of 15.3 ± 4.3. Prior to data collection, all participants signed a consent form approved by the KAIST IRB. Participants performed numerous practice swings until they felt comfortable with each club before commencing data collection. During data collection, participants first swung a driver 10 times, then a 7-iron 10 times. The preparation time between swings was approximately 10 s, and swings were initiated at the participant's own pace without specific instruction. Participants took a break for about 5 min before changing clubs. To synchronize the inertial sensor and the motion capture system, a light jump in place was performed prior to each swing.

**Figure 1:**
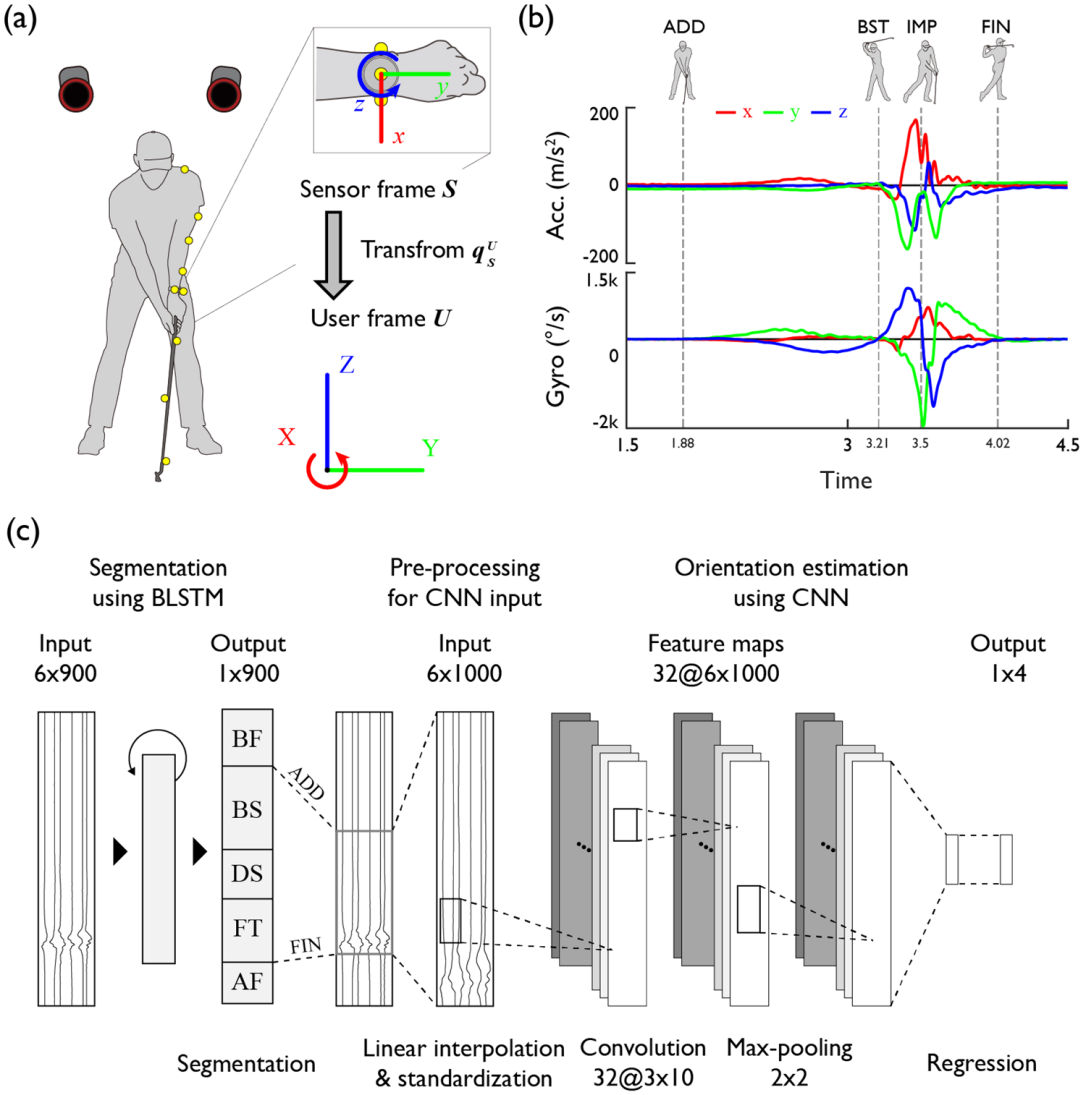
Experimental setup and signal processing overview. (**a**) Defining the sensor orientation using motion capture system and a wrist-worn IMU. (**b**) Acceleration and angular velocity signals of the IMU during the entire swing phases. (**c**) Segmentation, pre-processing, and orientation estimation of the inertial sensor signals.

During a swing, the kinematics of the upper body joints were recorded using both an inertial sensor and a motion capture system (Fig. 1). An IMU (BMA456, BMG250, Bosch Sensortec GmbH, Germany) affixed to the measured acceleration and angular velocity with a measurement range of ± 16 g, ± 2000 dps and a sampling frequency of 200 Hz (Fig. 1). In selecting the IMU, considering the feasibility of device applications such as smartwatches, we took into account the specifications of accelerometers and gyroscopes widely used in various commercial products and studies^52,53^. Optical markers were placed on the three planes of the inertial sensor (the top of the watch, the medial/lateral sides of the wrist), the left arm including the elbow and shoulder joints, the club, and the ball's position with respect to the club and ground. The positions were then captured using a motion capture camera (Hawk and Eagle, Motion Analysis, USA) with a sampling frequency of 200 Hz and an average 3D residual error of 1.0 ± 0.8 mm. The collected data were filtered at a cut-off frequency of 20 Hz using a Butterworth 10th order low-pass filter. The order and cut-off frequency of the filter were selected to preserve the significant power of the acceleration and angular velocity signal for the fast motion of the golf swing while minimizing the power and peak loss due to filtering^9,54–57^. Cubic spline interpolation was performed using ten sample data points before and after the clipping segments from the original IMU data for correcting the clipping. Post-processing was conducted to align the time axis of the inertial sensor and motion capture data by matching the peak instances of acceleration signals that occurred during a gentle on-the-spot jump prior to the swing. Out of the total 400 swings—10 trials each for two clubs conducted by 20 participants—we utilized data from 389 swings, excluding 11 instances where data collection errors occurred.

To segment the main sequences of a golf swing from the sensor signal, we employed a machine learning algorithm that trains on time-series data including features capable of identifying these sequences. Four main event points of a golf swing are defined by the movement of the club head: ADD, BST, IMP, and FIN (Fig. 1b). The ADD point is defined as the point where the club head velocity reaches a local minimum just before the backswing. The BST point is identified as the point after the backswing when the club head velocity passes through a local minimum. The IMP point is marked by the point during the downswing when the club head crosses the golf ball's Y-axis position. The FIN point corresponds to the point in the follow-through action when the club head velocity again passes through a local minimum. Following a previous study that showed higher segmentation accuracy when using a BLSTM^21^, this study also used a BLSTM to train on time series data from the wrist-worn IMU to segment the main swing phases (Fig. 1). For validation, the main swing sequences measured by an optical motion capture system were designated as the reference and compared.

### Estimation of sensor orientation

For convenience of player, we propose a method to estimate the sensor orientation with respect to the earth coordinate without the need for additional sensors or calibration actions. To compute the wrist trajectory from the data collected by the wrist-attached sensor, it is necessary to identify a quaternion *q*_***S***_^**U**^ that represents the rotation from the sensor's relative coordinate system, or sensor frame ***S***, to the ground-fixed absolute coordinate system, or user frame ***U*** (Fig. 1). Initially, the rotation quaternion is specified at the address as *q* (*t*_ADD_), which is the starting point of the swing data set. Subsequently, the rotation quaternion, or the sensor orientation for the entire swing segment can be computed by integrating the 3-axis angular velocity measured by the inertial sensor's gyroscope.

Firstly, to specify the rotation quaternion at the address, it is necessary to collect data that includes the sensor's orientation information. This research presumes that consistent sensor orientation can be extrapolated from specific signal segments around the address phase. This presumption is based on the observation that due to physical constraints, the change in the coordinate axis is relatively minor both within and between golfers (inter and intra-player) during the motion preceding and following the address^58–60^. The correlation between potential features that connote orientation information within the IMU data and the rotation quaternion at the address phase was trained using a CNN. To identify the most effective input data segment for training, we fed various combinations of data, ranging from the pre-address phase to major swing points such as the backswing top, impact, and finish, into the network. This includes static data segments preceding the address phase used in prior studies for orientation correction^25,27^. To validate the proposed method of sensor orientation estimation, it is necessary to define a reference for calculating absolute error and a baseline for comparison against the single inertial sensor-based orientation estimation method. The reference was defined by the quaternion value calculated by rotating the coordinate system of the inertial sensor, to which three optical markers were attached, to the ground coordinate system, as measured by the motion capture system for the corresponding trial of the subject. The baseline was statistically determined as the average quaternion values at the address point, measured from the motion capture data of all participants excluding the subject in question. Statistical significance for comparison validation was confirmed using a Student's t-test.

The preprocessing and model configuration of the CNN input values for quaternion estimation at the ADD are as Fig. 1. Preprocessing procedures were applied to employ subject-specific swing data as input values for the CNN. To adjust the magnitudes of the six IMU input data to similar levels, the magnitudes were standardized by their means and deviations calculated from other participants. The structure of the CNN consists of two convolutional layers, a max-pooling layer, and a fully connected layer, with each convolutional and max-pooling layer followed by a hyperbolic tangent activation function. The input values are a 6 × 1000 matrix consisting of 3-axis acceleration and 3-axis angular velocity from the inertial sensor, and the output values are a 1 × 4 size unit quaternion representing the sensor orientation at the ADD.

We utilized one of the metrics representing quaternion distance as our loss function, $\phi$, as

1 $${\varvec{\phi}}\left({{\varvec{q}}}_{{\varvec{t}}{\varvec{r}}{\varvec{u}}{\varvec{e}}},{{\varvec{q}}}_{{\varvec{e}}{\varvec{s}}{\varvec{t}}}\right)=1-\left|{{\varvec{q}}}_{{\varvec{t}}{\varvec{r}}{\varvec{u}}{\varvec{e}}}\cdot {{\varvec{q}}}_{{\varvec{e}}{\varvec{s}}{\varvec{t}}}\right|$$

which is the most direct and least computationally intensive approach amongst various metrics for quantifying the distance^61^ between the authentic quaternion ${q}_{true}$ of sensor orientation, ascertained through motion capture, and the estimated quaternion ${q}_{est}$, derived from the convolutional neural network. In quaternion notation, the quaternions $q$ and $-q$ denote the same rotation, regardless of their sign. Therefore, we constrained the sensor orientation that serves as the label of the output value of the CNN to reside in the northern hemisphere of the quaternion, where the scalar term is greater than or equal to zero. For the final validation of the trained model's performance, we employed a leave-one-out cross-validation method for the entire dataset, ensuring it did not include data from the subjects used in the training. After excluding the subject in question from the dataset as the test dataset, we randomly selected 90% of the total training dataset for training and assigned the remaining 10% as the validation set. We utilized a validation-based early stopping technique to prevent overfitting. The maximum number of epochs during training was set to 400. The Adam (adaptive moment estimator) optimization method^62^ was used as the optimization method.

Directly following the address, the initial orientation of the IMU at the commencement of a natural golf swing can be established through our previously described orientation estimation technique. Utilizing this initial value, we calculated the orientation of the IMU sensor frame relative to the user frame throughout the entire swing by integrating gyro signals over time. Then, by transforming IMU acceleration measured in the sensor frame into the user frame, we were able to integrate and compute the trajectory of the wrist. As a secondary step after specifying the quaternion at the ADD, the conversion of the coordinate system throughout the entire swing requires computing the rotational quaternion utilizing the 3-axis angular velocity signal captured from the gyroscope. Let the sampling time interval be $\Delta t$, and at time $t$, let the quaternion and angular velocity be $q(t)$, $\omega (t)$, respectively, the quaternion $q(t+1)$ at the next sampling time is calculated as

2 $${\varvec{q}}\left({\varvec{t}}+1\right)={\varvec{q}}\left({\varvec{t}}\right)+\frac{1}{2}\left({\varvec{q}}\left({\varvec{t}}\right)\otimes{\varvec{\omega}}\left({\varvec{t}}\right)\right)\times{\varvec{\Delta}}{\varvec{t}}$$

where $\otimes$ denotes a quaternion product. When calculating the quaternion of the swing interval based on the inertial sensor, drift errors inevitably occur due to the accumulation of noise in the angular velocity of the gyro. To examine the contribution of the proposed orientation estimation method at the ADD to the error reduction during orientation calculation throughout the swing, we made comparisons with scenarios where the orientation specification at the ADD was employed as a reference and baseline. The true sensor orientation across the entire swing phase were determined using an optical motion capture camera system. On the other hand, the baseline was defined as a method that can be calculated using only IMU signals, assuming the availability of reference golf swing data. Specifically, the baseline was defined as the orientation calculated by integrating the IMU angular velocity for the swing phase, with the initial orientation set as the average of the ground truth orientations at the address position of all subjects except the subject in question. The coordinate error was quantified as the rotation angle, which was calculated using the axis-angle representation of the quaternion difference. To ensure a limited range of the error angle ${\theta }_{{\text{error}}}$ within $-\uppi <{\theta }_{{\text{error}}}\le \pi$, atan2 function was employed.

### Reduction of drift error

In order to compute the swing trajectory from a single IMU affixed to the wrist, it is necessary to perform double integration of the acceleration signals for each axis in the absolute coordinate system, as delineated in the preceding section on orientation estimation. Drift errors are inevitable during this integration process due to noise accumulation within the IMU signals. Therefore, this study presents a method aimed at mitigating these drift errors, leveraging the kinematic constraints inherent to wrist movement during a golf swing.

**Figure 2:**
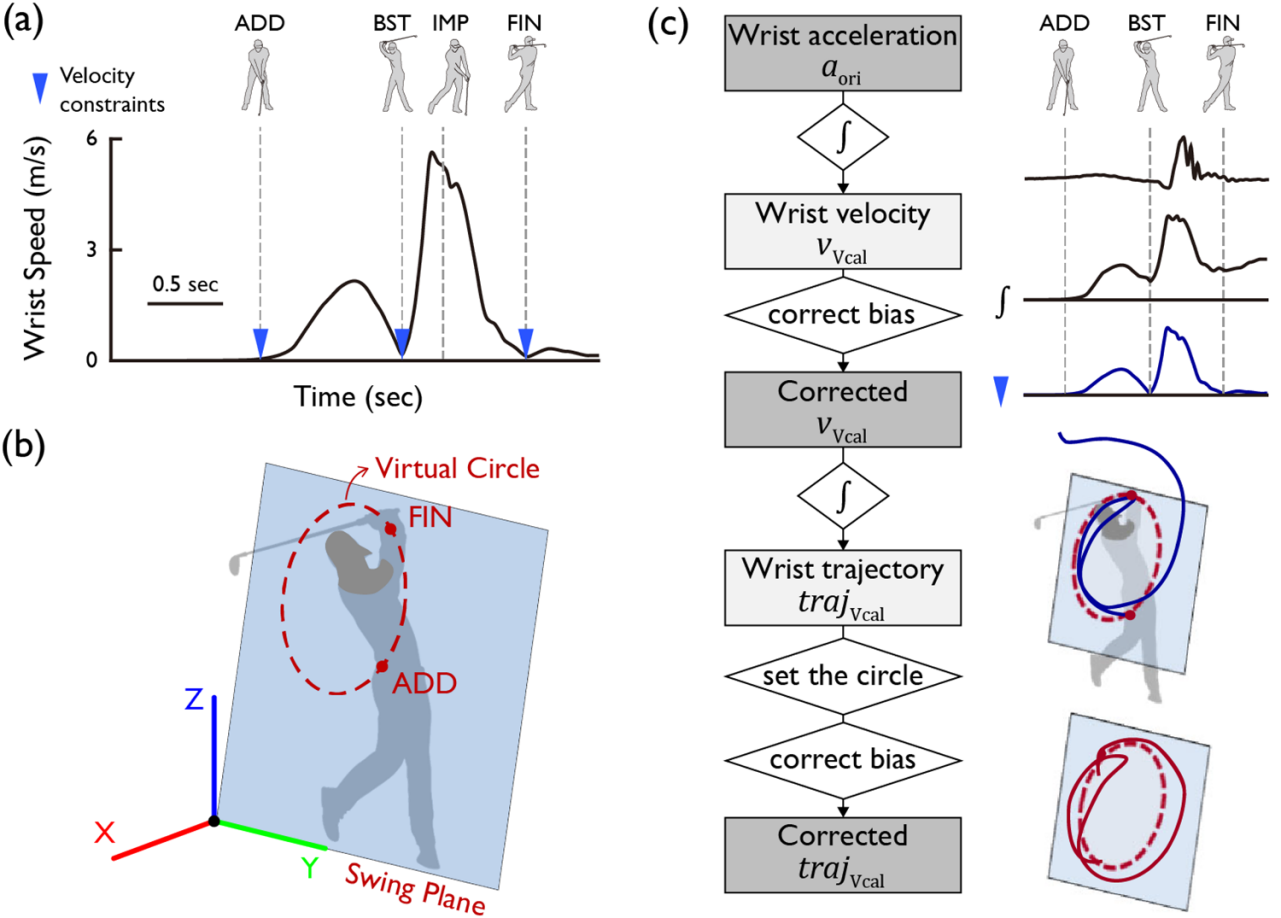
A schematic of the drift error reduction method. (**a**) Characteristics of the left wrist velocity approximating zero at the ADD, BST, and FIN points during the swing. (**b**) Characteristics of the left wrist trajectory lying on a virtual circle in the swing plane. (**c**) Velocity and trajectory correction methods using kinematic constraints of the wrist.

To eliminate the drift error of the velocity that occurs when integrating the acceleration, we applied a constraint that stipulates the wrist speed at the ADD, BST, and FIN approximates zero (Fig. 2). We posited that a time-based linear function could eliminate the drift error occurring during integration, leading us to adopt the subsequent time-based linear correction. The original velocity ${v}_{i,{\text{ori}}}$ in X–Y–Z ground-fixed coordinate is calculated by the integration of the original acceleration ${a}_{i,{\text{ori}}}$ as

3 $${v}_{i,{\text{ori}}}\left(t\right)={\int }_{{t}_{{\text{ADD}}}}^{t}{a}_{i,{\text{ori}}}\left(t\right)dt, \left({t}_{{\text{ADD}}}\le t\le {t}_{{\text{FIN}}}, i={\text{X}},{\text{Y}},{\text{Z}}\right).$$

After applying the time-based linear correction, calibrated velocity ${v}_{i,\mathrm{ Vcal}}$ from ${t}_{{\text{ADD}}}$ to ${t}_{{\text{BST}}}$ is calculated as

4 $${v}_{i,\mathrm{ Vcal}}\left(t\right)={v}_{i,{\text{ori}}}\left(t\right)-{v}_{i,{\text{ori}}}\left({t}_{{\text{BST}}}\right)\times \frac{t-{t}_{{\text{ADD}}}}{{t}_{{\text{BST}}}-{t}_{{\text{ADD}}}}, \left({t}_{{\text{ADD}}}<t\le {t}_{{\text{BST}}}, i={\text{X}},{\text{Y}},{\text{Z}}\right),$$

and ${v}_{i,\mathrm{ Vcal}}$ from ${t}_{{\text{BST}}}$ to ${t}_{{\text{FIN}}}$ is calculated as

5 $${v}_{i,\mathrm{ Vcal}}\left(t\right)={v}_{i,{\text{ori}}}\left(t\right)-{v}_{i,{\text{ori}}}\left({t}_{{\text{BST}}}\right)-{v}_{i,{\text{ori}}}\left({t}_{{\text{FIN}}}\right)\times \frac{t-{t}_{{\text{BST}}}}{{t}_{{\text{FIN}}}-{t}_{{\text{BST}}}}, ({t}_{{\text{BST}}}<t\le {t}_{{\text{FIN}}}, i={\text{X}},{\text{Y}},{\text{Z}}).$$

To mitigate the drift error that arises when integrating velocity to obtain trajectory, we exploited the feature that the trajectory of the left wrist during a swing closely mirrors a circle on the swing plane^5,63^ (Fig. 2). In particular, a virtual circle was established on the 3D swing plane, and a constraint was implemented stipulating that the swing trajectory remains within this virtual circle. The 3D swing plane was defined by the normal vector ${\overrightarrow{{\text{v}}}}_{{\text{n}}}$ of the plane that yields the minimum sum of squared orthogonal distances from the wrist trajectory data during the backswing, using singular value decomposition. The center ${{\text{c}}}_{{\text{circ}}}$ and radius ${{\text{r}}}_{{\text{circ}}}$ of the virtual circle were calculated using the least squares method after projecting the wrist displacement data onto the swing plane. The wrist trajectory ${Traj}_{i,{\text{Vcal}}}$ was calculated from the corrected velocity ${v}_{i,\mathrm{ Vcal}}$ as

6 $${Traj}_{i,{\text{Vcal}}}\left(t\right)={\int }_{{t}_{{\text{ADD}}}}^{t}{v}_{i,{\text{Vcal}}}\left(t\right)dt, \left(i={\text{X}},{\text{Y}},{\text{Z}}\right).$$

We then corrected the bias error in the acceleration signal in the x–y–z sensor coordinate system to satisfy the constraint that the displacement at the FIN, where the drift error accumulates the most during integration, is located on the virtual circle (Fig. 2). We can calculate the difference ${{\text{d}}}_{{\text{FIN}}}$ between ${Traj}_{i,{\text{Vcal}}}\left({t}_{{\text{FIN}}}\right)$ and the swing plane as

7 $${{\text{d}}}_{{\text{FIN}}}=\left(\left({Traj}_{i,{\text{Vcal}}}\left({t}_{{\text{FIN}}}\right)-{{\text{c}}}_{{\text{circ}}}\right) \cdot {\overrightarrow{{\text{v}}}}_{{\text{n}}}\right){\overrightarrow{{\text{v}}}}_{{\text{n}}}.$$

Then, ${Traj}_{i,{\text{Vcal}}}\left({t}_{{\text{FIN}}}\right)$ can be projected to the swing plane as

8 $${{\text{r}}}_{{\text{cal}}}={Traj}_{i,{\text{Vcal}}}\left({t}_{{\text{FIN}}}\right)-{{\text{d}}}_{{\text{FIN}}}.$$

The endpoint of the trajectory can be corrected and located on the virtual circle at ${\text{ep}}$, calculated as

9 $${\text{ep}}={{\text{c}}}_{{\text{circ}}}+{{\text{r}}}_{{\text{circ}}}\left(\frac{{{\text{r}}}_{{\text{cal}}}-{{\text{c}}}_{{\text{circ}}}}{\Vert {{\text{r}}}_{{\text{cal}}}-{{\text{c}}}_{{\text{circ}}}\Vert }\right).$$

From the corrected endpoint ${\text{ep}}$, the local acceleration bias ${a}_{j,{\text{bias}}}$ in x–y–z coordinate is estimated as

10 $${a}_{j,{\text{bias}}}={\left({Traj}_{{\text{Vcal}}}\left({t}_{{\text{FIN}}}\right)-{\text{ep}}\right)}_{j}\times \frac{1}{{\Delta t}^{2}}, (j={\text{x}},{\text{y}},{\text{z}}).$$

By subtracting ${a}_{j,{\text{bias}}}$ from ${a}_{j,{\text{Vcal}}}$, which is differentiated by $t$ from ${v}_{j,{\text{Vcal}}}$ in x–y–z coordinate, ${a}_{j,{\text{Tcal}}}$ is calculated as

11 $${a}_{j,{\text{Tcal}}}\left(t\right) ={a}_{j,{\text{Vcal}}}\left(t\right)- {a}_{j,{\text{bias}}}, \left({t}_{{\text{ADD}}}\le t\le {t}_{{\text{FIN}}}, j={\text{x}},{\text{y}},{\text{z}}\right).$$

Finally, the corrected trajectory ${Traj}_{i,{\text{Tcal}}}$ is calculated such that the endpoint is located on the virtual circle as

12 $${Traj}_{i,{\text{Tcal}}}\left(t\right)={\iint }_{{t}_{{\text{ADD}}}}^{t}{a}_{i,{\text{Tcal}}}\left(t\right)dt, \left({t}_{{\text{ADD}}}\le t\le {t}_{{\text{FIN}}}, i={\text{X}},{\text{Y}},{\text{Z}}\right).$$

As a final step, we confirmed the accuracy of the wrist swing trajectory derived from a single IMU by sequentially applying the orientation estimation method coupled with the trajectory correction method proposed in this study. A paired t-test was conducted to verify that the error of our proposed IMU signal processing method was significantly lower than that of existing methods, using the ground truth data from motion capture as the reference.

In this study, we calculated and compared the errors between the proposed method and existing methods based on the values measured by the optical motion capture camera for angle, velocity, and trajectory. For the orientation angle error in the address and entire swing phases, we used the angle between the quaternions representing the rotation of the sensor frame in the user frame, expressed in the axis-angle representation. For velocity and trajectory, we used the mean absolute error (MAE) for each of the X–Y–Z axes, or the 3D vector MAE, as the error metrics.

## Results and discussion

### Segmentation of sensor data

By applying BLSTM to time-series data from the wrist-worn inertial sensor, we extracted signals corresponding to the golf swing phase, with an average segmentation error of 38 ± 19 ms (mean ± SD). The errors at the ADD and FIN points (61–69 ms) were approximately five times larger than those at the BST and IMP points (9–15 ms). This is consistent with previous studies due to the low signal-to-noise ratio employed for swing sequence identification at the beginning and the end of the swing phase^21,64^.

### Estimation of sensor orientation

**Figure 3:**
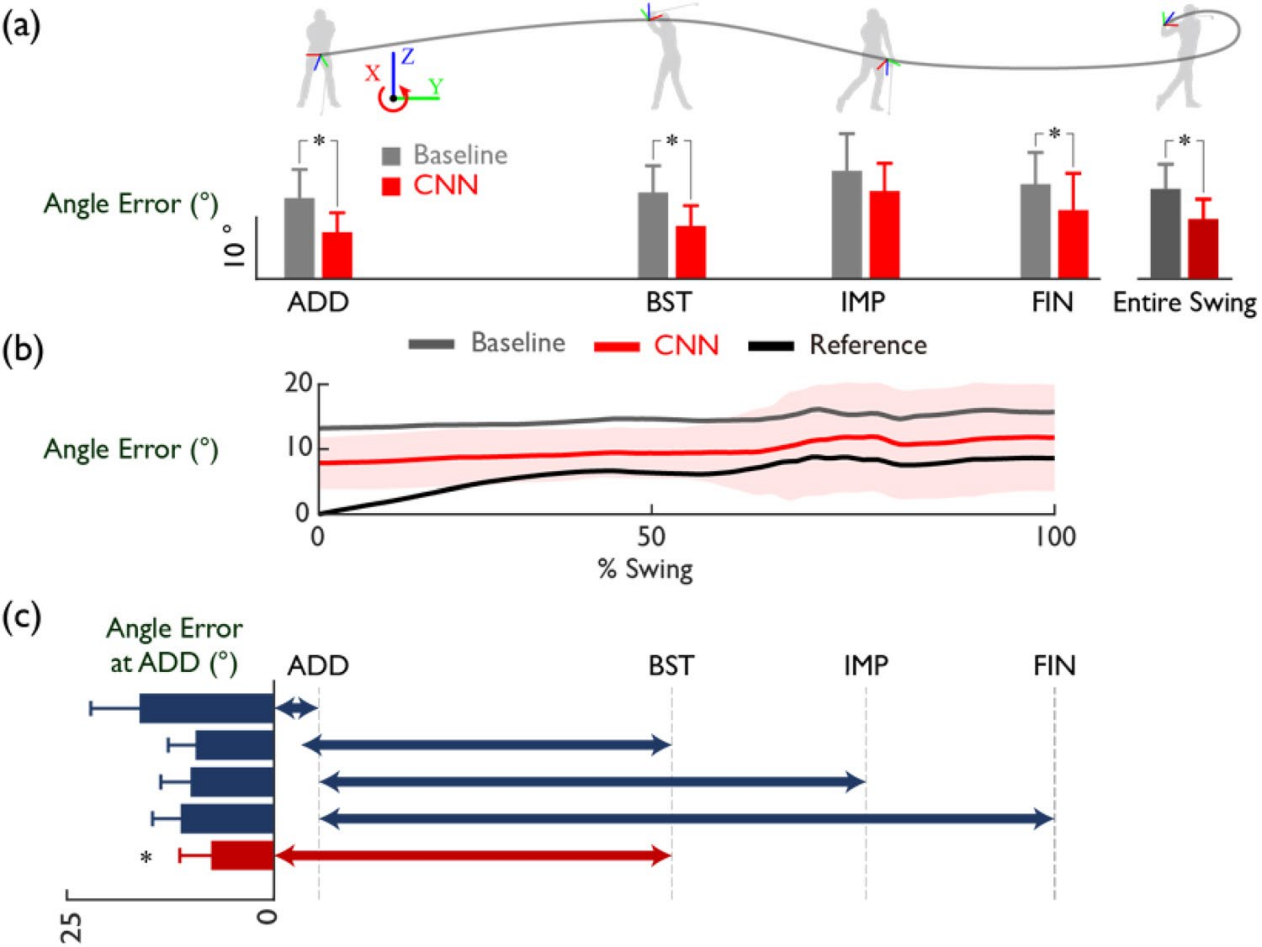
Validation results of the proposed CNN-based sensor orientation estimation method. (**a**) Angle errors of the orientation estimation at ADD, orientation calculation at BST, IMP, FIN, and for the entire swing phases (*p < 0.05). (**b**) Comparison of orientation errors in the entire swing phases based on different methods for orientation estimation at ADD. The shaded area represents the standard deviation of the CNN method. (**c**) Comparison of orientation estimation errors for ADD based on different input ranges of CNN. The range before ADD is 100 ms in length (*p < 0.05 compared to other methods).

By implementing the proposed sensor orientation estimation method to the wrist-worn IMU data during a natural golf swing, we achieved an error level of 133% compared to the reference and 63% compared to the baseline. At the ADD and throughout the entire swing phase, the mean sensor orientation errors are 7.6 ± 3.1° and 9.5 ± 3.2° respectively (mean ± SD) (Fig. 3). The sensor orientation error increases over time due to drift error from angular velocity integration. Particularly, the error and deviation escalate dramatically around the moment of impact, where the value of angular velocity increases abruptly (Fig. 3). The findings from the proposed method reveal a similar degree of error to those found in the previous research that applied widely used calibration poses by the subjects such as the N-pose and T-pose^26^. In this study, we did not perform orientation calibration using N-pose or T-pose data, which seems to have limitations in applying to the actual golf field, because our ultimate goal was to overcome the technical hurdles of monitoring swing trajectory using smartwatches during natural golf play in the field. However, similar to the N-pose and T-pose calibration method, which utilizes known orientation information during static calibration movements for IMU orientation estimation, this study applied the initial static pose information during address to orientation estimation, achieving a similar level of calibration accuracy as the hand orientation calibration using N-pose and T-pose^26^. Also, in comparison with several studies that used inertial sensors to estimate orientation or joint angles (Table 1), our approach displays an error level of similar magnitude.

**Table 1:** Prior studies on orientation estimation and calibration of IMUs in various domains of human motion monitoring.

Author	Ueda^65^	Falbriard^66^	Zimmermann^42^	Adamowicz^67^	McGrath^68^	Shin^69^
Angle error	11° per axis	5–9° standard deviation	8–21° per axis	8–12°	4.3–14.5° per axis	6–8°
Sensor	1 IMU	1 IMU	7 IMUs	8 IMUs	8 IMUs	13 IMUs
Initial cal	Mocap	standing	N-pose	standing	cal. motion	none
Motion	golf swing	running	walking	walking	walking	various

*In most cases, especially when using a small number of sensors, specific actions for the initial calibration are needed.

It is worth noting that our proposed method demonstrated the feasibility of correcting the orientation of an IMU sensor for a fast and wide range of 3D rotational motion like golf swing. The maximum acceleration and angular velocity observed in the wrist-hand during the golf swings measured in this study were about 111 ± 31 m/s^2^ and 1627 ± 230°/s, respectively. These are considerably faster than the dynamic conditions used for previous studies regarding IMU orientation correction such as 5°/s^34^, 180–360°/s^35^, 110–240°/s^70^, 800–970°/s^71^. Furthermore, the rotation of the wrist during golf swing demonstrates a wide 3D rotational motion ranged from 150° to 350° in the x–y–z axes. Contrarily, previous investigations into IMU-based motion tracking have predominantly focused on actions such as walking, jogging, and squatting, which exhibit a rotational motion span of about 10° to 60° across each respective axis^42,67–69^. Fast movements with wide 3D rotation measured with an IMU result in large measurement and the drift error^27^. Despite the challenges, we successfully estimated the sensor orientation with an error level comparable to those of previous studies, highlighting the effectiveness of our proposed method. The satisfactory calibration results of the IMU orientation during natural golf swings using the CNN suggest that features related to coordinate orientation information can be derived from the time-series data of a wrist-worn IMU. Meanwhile, we adopted a quaternion integration method (Eq. 2) for calculating sensor orientation based on angular velocity from IMU, which is the simplest and computationally cost-effective method and has been widely used in previous studies^34,72,73^. On the other hand, there have been reports of cases where the orientation error was reduced by applying higher-order integration methods such as the fourth-order Runge–Kutta approach to dynamic state^74^. Therefore, it would be meaningful to conduct future studies that combine the error reduction method proposed in this study with higher-order integration methods to reduce the swing trajectory estimation error of high-speed movements such as golf swings.

One thing to consider when evaluating the results of this study is the potential error in the orientation training data. The orientation values used as ground truth for training the CNN model for orientation estimation were measured using three optical markers attached to a watch-shaped wearable device. According to Yang et al., the static angle error calculated from closely spaced markers (13–24 mm apart) is approximately 0.21–0.39°^75^. In this study, the average distance between the optical markers fixed on the watch was approximately 69.2 ± 2.3 mm, which is larger than the spacing in the previous study. Therefore, the error caused by interference from closely spaced markers is likely to be similar to or lower than that in the previous study. However, even assuming that such an error occurs, the average error improvement achieved by the proposed orientation estimation method in this study is 4.5°. Therefore, it can be said that the statistical significance of this estimation is maintained even when considering the error caused by closely spaced markers.

It is interesting to note that using orientation information from the dynamic phase between address and backswing top was more effective for orientation estimation than using only the static address orientation information (Fig. 3). The most effective IMU data for training the CNN to estimate sensor orientation at the ADD includes both the static motion data prior to the ADD and the dynamic data up to the BST as illustrated in Fig. 3. Similar to static IMU calibration poses such as the N-pose and T-pose, gravitational acceleration is used to estimate orientation in the Z-axis when IMU data during the static motion period is input into the CNN. Nonetheless, this segment's limited acceleration information on the X and Y axes hinders the calibration of their corresponding orientation, leading to a relatively lower calibration accuracy. The addition of the data segment from the ADD to the BST into the CNN training enhances calibration accuracy by providing extra information about the X–Y–Z-axis orientation. However, the estimation error rises when downswing data, characterized by a dramatic change in wrist speed direction, is included. This suggests that the most effective orientation calibration employs data from phases exhibiting comparatively consistent and smooth wrist coordinate system rotation. It is also noteworthy that by learning the orientation information of the IMU during someone else's address, the subject can calibrate the orientation of the inertial sensor with high accuracy without performing any prior calibration movements. This implies that there are common features in the IMU signal collected from the address to the backswing top of the golf swing that are necessary for orientation estimation. However, it can also be interpreted that the swing data of the golfers who participated in this study had high consistency. In this case, if there is a large variation in swing motion between golfers of different skill levels, the accuracy of the calibration method proposed in this study may decrease and further consideration is needed.

Training the CNN model with a combined dataset encompassing swing data from two different subject groups and two types of clubs yielded lower estimation errors than training the model with each group separately (Table 2). Previous studies have noted between-club differences for golf swing kinematics^76,77^, and discrepancies have also been observed in swing maneuvers between professional and amateur golfers^78–80^. Nonetheless, when we separately divided swing samples generated with a driver and a 7-iron for training and validation, the orientation estimation error at the address position exhibited greater average discrepancies (8.6 ± 4.4° and 9.6 ± 5.2° respectively), compared to the approach of merging the swing datasets from both clubs for training. One potential explanation for this could be the existence of common features in the wrist-worn IMU signals that are valuable for orientation estimation training of the CNN, regardless of the variations across subject groups and club types. Another plausible explanation, and a limitation of our study, is that the data volume used for CNN training might have been insufficient to accurately contrast calibration performance across different subject groups and clubs. The roughly 200 swing trials conducted for each club in this study may not provide an adequately large dataset to ensure the convergence of the CNN model. Du et al. have demonstrated that even within a data sample range of 1,000 to 2,000, a simple CNN model with linear activation functions can consistently decrease in error^81^. Consistent with prior studies utilizing CNN models^82,83^, we anticipate that incorporating additional swing data into training could enhance accuracy and enable the comparison of calibration results, which may vary by golf expertise or club types.

**Table 2:** Comparison of sensor orientation estimation and calculation results for different groups of training datasets in CNN, with mean values and standard deviations (mean ± SD) (*p < 0.05 compared to other groups).

Swing event points		ADD	BST	IMP	FIN	Entire swing
Total		*7.6 ± 3.1°	8.5 ± 3.3°	14.1 ± 4.5°	11.1 ± 5.9°	9.5 ± 3.2°
Skill level	Professional	9.4 ± 3.9°	10.1 ± 4.0°	15.0 ± 2.8°	13.2 ± 5.1°	11.3 ± 3.5°
	Amateur	9.7 ± 3.8°	10.4 ± 3.1°	12.6 ± 5.0°	13.6 ± 5.6°	11.3 ± 3.6°
Club	Driver	8.6 ± 4.4°	9.1 ± 4.5°	14.9 ± 5.9°	10.6 ± 5.7°	10.0 ± 3.9°
	7-iron	9.6 ± 5.2°	10.8 ± 4.9°	15.4 ± 6.3°	13.6 ± 7.5°	11.9 ± 5.0°

### Reduction of drift error

**Figure 4:**
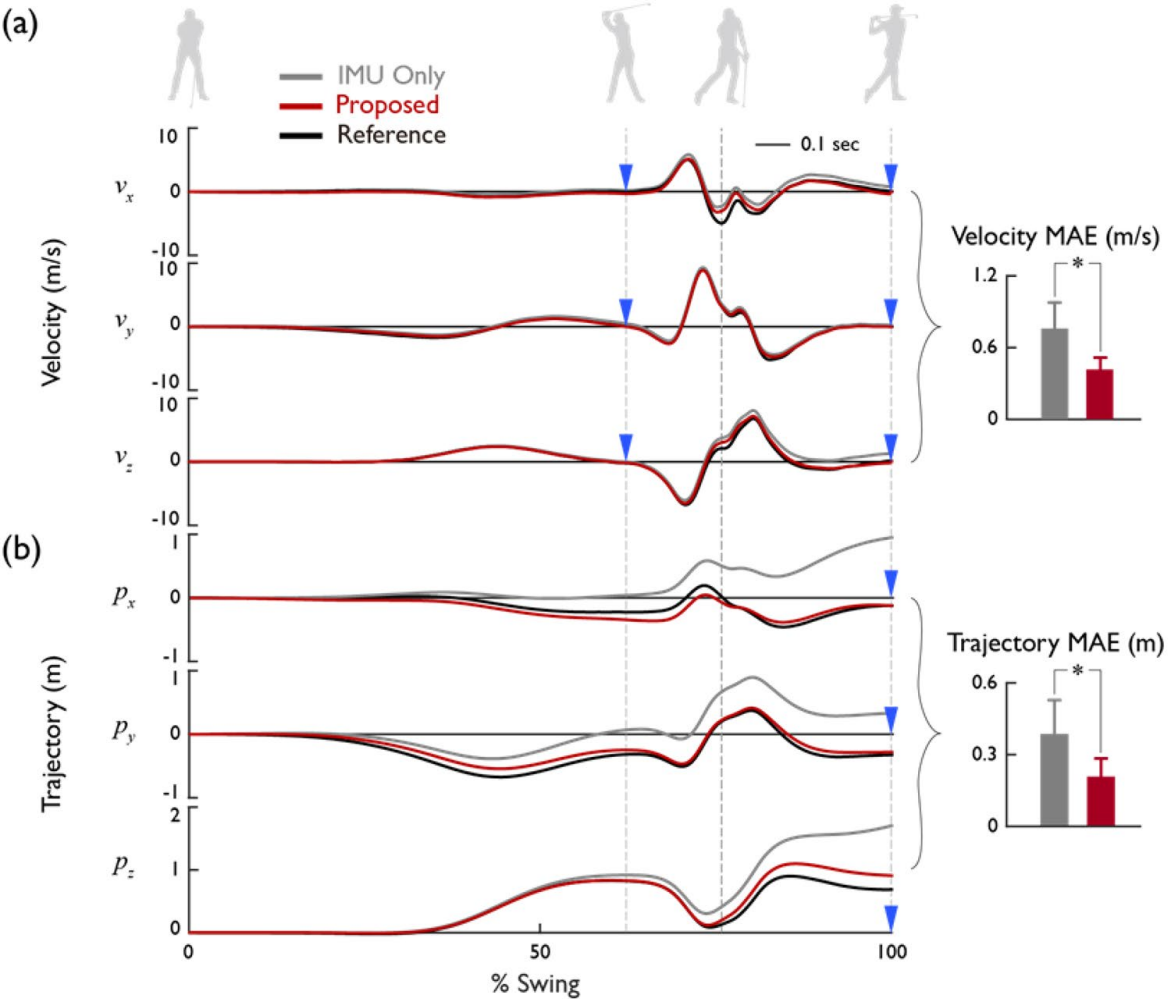
Validation results of the proposed drift error reduction method. A comparison of the (**a**) velocity and (**b**) trajectory calculations using only IMU and applying the proposed method for a representative swing trial, considering the ground-fixed X, Y, and Z axes. Blue inverted triangles indicate the points where kinematic characteristics are reflected. The bar graph compares the mean absolute error values based on the norm values (*p < 0.05).

We confirmed the validity of the two assumptions set for wrist trajectory correction: zero velocity approximation and the circular trajectory of wrist. Our proposed method for reducing drift error contributed to a significant enhancement in wrist trajectory accuracy. The velocities at the ADD, BST, and FIN relative to the maximum swing speed were 0.9 ± 1.2%, 4.8 ± 1.9%, and 2.1 ± 0.9% respectively, and are substantially approximating to zero. The R-squared value between the wrist trajectory from the motion capture and the fitted virtual circle was 0.984 ± 0.007 across the whole swing phase, confirming the validity of the circular trajectory assumption (Table 3). Utilizing the proposed trajectory estimation method led to a significant reduction (p = 3e−5) of more than 40% in the absolute velocity and trajectory errors, from 0.76 ± 0.22 m/s to 0.42 ± 0.10 m/s, and from 0.39 ± 0.14 m to 0.21 ± 0.08 m, respectively (Fig. 4). This outcome aligns with previous studies^30,31^ that mitigated integration errors through the application of ground contact points of the foot during walking. This suggests that the kinematic properties of motion can contribute to enhancing the accuracy of IMU-based motion monitoring.

**Table 3:** Comparison of the circular trajectory assumption quantitatively using R-squared value between the wrist trajectory obtained from the motion capture and the fitted virtual circle for different groups, with mean values and standard deviations (mean ± SD).

Swing phases		Backswing	Downswing	Follow-through	Entire swing
Total		0.991 ± 0.005	0.944 ± 0.028	0.957 ± 0.023	0.984 ± 0.007
Skill level	Professional	0.992 ± 0.002	0.960 ± 0.016	0.949 ± 0.020	0.984 ± 0.005
	Amateur	0.990 ± 0.006	0.930 ± 0.028	0.964 ± 0.024	0.984 ± 0.009
Club	Driver	0.991 ± 0.005	0.927 ± 0.038	0.951 ± 0.025	0.981 ± 0.008
	7-iron	0.992 ± 0.004	0.961 ± 0.019	0.962 ± 0.023	0.986 ± 0.007

The proposed wrist trajectory estimation method considerably improved wrist trajectory estimation despite differences in swing patterns between clubs. In the driver swing, the mean absolute trajectory error decreased by approximately 40%, from 0.350 ± 0.129 m to 0.213 ± 0.079 m, and in the 7-iron swing, it decreased by approximately 52%, from 0.424 ± 0.162 m to 0.203 ± 0.077 m (Table 4). Prior to the application of the drift error reduction method, the 7-iron swing showed a larger mean absolute error in both speed and trajectory in comparison with the driver swing. However, after error correction, no considerable differences were found in both speed and trajectory across clubs. The 7-iron swing exhibited a more substantial correction effect as it more closely conformed to the circular approximation assumption of the wrist trajectory in the swing plane (Table 3).

**Table 4:** Comparison of velocity and trajectory errors before (IMU only) and after (proposed) applying the suggested drift error reduction method for different groups, with mean values and standard deviations (mean ± SD) (*p < 0.05 compared to IMU only).

		Velocity MAE (m/s)		Trajectory MAE (m)	
methods		IMU only	Proposed	IMU only	Proposed
Total		0.760 ± 0.217	*0.415 ± 0.103	0.387 ± 0.141	*0.208 ± 0.076
Skill level	Professional	0.929 ± 0.164	*0.464 ± 0.101	0.504 ± 0.096	*0.228 ± 0.067
	Amateur	0.621 ± 0.144	*0.376 ± 0.086	0.292 ± 0.090	*0.191 ± 0.078
Club	Driver	0.694 ± 0.212	*0.402 ± 0.088	0.350 ± 0.129	*0.213 ± 0.079
	7-iron	0.826 ± 0.285	*0.428 ± 0.131	0.424 ± 0.162	*0.203 ± 0.077

The trajectory estimation method exhibited its most substantial effect around the FIN. The drift error, which accumulates due to the integration process, escalates over time, with a pronounced increase observed near the IMP, where the swing speed is at its maximum. Consequently, the greatest error is shown at the finish of the swing (Fig. 4, Table 5). By incorporating a constraint that posits the wrist trajectory at the FIN to lie on a virtual circle within the swing plane, we were able to effectively rectify the substantial drift error observed at the FIN. The most substantial velocity and trajectory errors were observed along the Y-axis, which is the progression direction of the swing (Table 6). Large errors in the Y-direction may be attributed to the high Y-axis speed near the IMP point and the absence of velocity or trajectory adjustment constraints around the IMP. Therefore, to improve this, additional consideration is needed to ascertain if there is kinematic information that can be utilized for trajectory correction near the IMP.

**Table 5:** Comparison of velocity and trajectory errors before (IMU only) and after (proposed) applying the suggested drift error reduction method, based on swing phases, with mean values and standard deviations (mean ± SD) (*p < 0.05 compared to IMU only).

Swing phase	Backswing		Downswing		Follow-through	
Methods	IMU only	Proposed	IMU only	Proposed	IMU only	Proposed
Velocity MAE (m/s)	0.287 ± 0.092	0.270 ± 0.093	0.662 ± 0.247	*0.560 ± 0.142	1.630 ± 0.499	*0.611 ± 0.140
Trajectory MAE (m)	0.119 ± 0.046	0.112 ± 0.042	0.340 ± 0.123	*0.266 ± 0.123	0.863 ± 0.309	*0.343 ± 0.140

**Table 6:** Comparison of velocity and trajectory errors before (IMU only) and after (proposed) applying the suggested drift error reduction method, based on X–Y–Z coordinates, with mean values and standard deviations (mean ± SD) (*p < 0.05 compared to IMU only).

Axis	X		Y		Z	
Methods	IMU only	Proposed	IMU only	Proposed	IMU only	Proposed
Velocity MAE (m/s)	0.349 ± 0.152	*0.184 ± 0.074	0.485 ± 0.149	*0.261 ± 0.080	0.300 ± 0.133	*0.174 ± 0.044
Trajectory MAE (m)	0.178 ± 0.093	*0.079 ± 0.035	0.264 ± 0.129	*0.136 ± 0.072	0.134 ± 0.070	*0.095 ± 0.049

We proposed an IMU integral error reduction method utilizing kinematic information, such as trajectory changes induced by arm joint movements during a golf swing. In addition, there is a possibility that information that can be used for error correction exists if we analyze the wrist orientation trajectory during the swing, even though it is relatively difficult to visualize. For instance, we suggest, as a potential avenue for future research, the utilization of quantitative characteristics such as the gradual rotation of wrist orientation in the early phase of the backswing or the symmetry around the backswing top, for error reduction.

### Validation of the proposed methods

When we combined the orientation estimation and drift error reduction methods proposed in this study, the wrist trajectory error was reduced to 0.168 ± 0.054 m. This demonstrates a significant reduction (p = 2e−9) of approximately 82.3 ± 9.5%, compared to the baseline error of 1.094 ± 0.388 m (Table 7). The baseline error was calculated using statistical orientation and simple integration of the IMU signal. Moreover, to quantitatively assess the similarity between the estimated and reference trajectories in 3D space, we calculated the R-squared value to be 0.859 ± 0.081. This represents a substantial improvement compared to the baseline R-squared value of − 0.766 ± 0.313.

**Table 7:** Estimation errors of wrist swing velocity and trajectory using the baseline method for sensor orientation estimation and simple integration of acceleration (IMU only), and the proposed methods for sensor orientation estimation and drift error reduction in series, with mean values and standard deviations (mean ± SD) (*p < 0.05 compared to IMU only).

Methods	Velocity MAE (m/s)		Trajectory MAE (m)	
	IMU only	Proposed	IMU only	Proposed
Total	1.587 ± 0.471	*0.394 ± 0.127	1.094 ± 0.388	*0.168 ± 0.054

Despite using only single IMU data, the proposed trajectory estimation method demonstrated tracking performance comparable to golf swing tracking methods that additionally utilize multimodal data such as images. The full golf swing trajectory spans approximately 4.4 m, and we estimated the wrist trajectory with an average error of about 17 cm, yielding an error level of about 4% relative to the entire motion range. This error level aligns with those reported in prior golf tracking studies that employed vision sensors like depth or stereo cameras, either independently or in combination with an IMU. Jia et al. reported a wrist trajectory estimation error of 17 cm using a depth camera alone and roughly 10 cm when integrated with an IMU^11^. Nam et al. demonstrated a club trajectory estimation error of 13.2 cm by fusing data from an IMU and LED attached to the club with information from a ground-installed stereo camera^14^. However, Cheon et al. recorded an average error of 7 cm for clubhead trajectory estimation utilizing a single IMU, showcasing a higher trajectory accuracy compared to our study, thus warranting further exploration^18^. The single IMU-based trajectory tracking method introduced in this study is noteworthy due to the convenience and data utility it ensures in contrast to video-based tracking techniques.

When compared with prior motion tracking studies using a single inertial sensor, the method proposed in this study effectively eliminated the cumulative error in rapid, non-periodic movements. Previous work on gait tracking employing a single inertial sensor has yielded estimation errors of 6–7%^32^ and 12–25%^84^ proportional to the range of motions, and errors around 28.1 cm for jumping movements^85^. By computing the foot trajectory during walking via an inertial sensor affixed to the instep, Kitagawa et al. estimated the stride to within an error margin of 5 cm (3–4% of stride length)^43^. Hao and colleagues estimated stride length and step width with RMS errors of 1.14 cm and 0.95 cm respectively, using an inertial sensor attached to the rear of the foot^86^. Cardarelli et al. used an inertial sensor attached to the sacrum to estimate the displacement of the body's center of gravity to within 0.25 cm during treadmill walking^32^. In the case of gait, the limb movements are coordinated, joint kinematics of many subjects are quite consistent, motions are periodic and under moderate speed, and there are repeated ZUPT moments. As a result, many prior studies employing single IMU-based trajectory tracking have reported high levels of accuracy. However, for golf swing tracking, which has large variability between players, skill levels, and clubs, and involves fast movements over a wide range of 3D rotation, single IMU-based trajectory tracking can yield high errors up to 30–60 cm^11^ (Fig. 4, Table 7). Taking into account the difficulties inherent in tracking golf swing motions, it is worth highlighting that our study has successfully mitigated tracking errors to a remarkable extent in movements that are rapid, non-periodic, and span a wide 3D rotational range.

We investigated whether the IMU used for measurement accurately measured the rapid kinematic information of the wrist during a golf swing. Among the 389 swings collected in the experiment, it was observed that 150 swings showed clipping around 0.9% of the time during the impact phase within an average swing duration of 2.14 s. To address this, we performed cubic spline interpolation using ten sample data points before and after the clipping segments from the original IMU data to correct the clipping. Through this correction, the peak error between the acceleration and angular velocity from the markers and the IMU decreased from 9.1 ± 6.4% to 7.4 ± 5.7% and 16.5 ± 10.5% to 7.3 ± 6.3%, respectively. To examine whether the use of clipping corrected IMU data affect to the swing trajectory correction method proposed in this study, we performed the trajectory correction using acceleration and angular velocity calculated from motion capture assuming that IMU data without clipping was used. In this case, swing trajectory error was reduced by approximately 78.3 ± 10.7%, which was not statistically significantly different (p > 0.05) from the error reduction using the clipping corrected IMU data in Table 7. Therefore, it can be said that the adopted clipping correction method has a limited effect on the main research results of the swing trajectory error reduction proposed in this study.

It is worth to note that the proposed orientation estimation and drift removal methods utilize the characteristic of consistency in swing motion observed across golfers. Therefore, the effectiveness of the proposed trajectory correction method may be reduced for golfers with lower swing consistency than those who participated in this study. However, previous studies by Brown et al.^87^, and Glazier et al.^88^ have reported that while golf swings exhibit high intra-individual (within-person swing) consistency, there is also large inter-individual (between-person) variability in the swings of even high-skill level golfers, making it difficult to find a common optimal technique. Glazier et al.^88^, Tucker^89^, and Langdown et al.^60^ argued that there is a lack of research on the criteria for individual and intra-individual movement variability in golf swings and whether this is beneficial or detrimental to swing performance, and that more research is needed on this complex topic. Therefore, future studies with swing data from golfers with different skill levels and swing styles are needed to investigate the potential for generalizing the results of this study.

In this study, we successfully tackled technical challenges to enhance the accuracy and convenience of golf swing tracking using a wrist-worn IMU. By harnessing observed kinematic characteristics from golfers, including consistent and continuous sensor coordinate rotation around the ADD, and by incorporating machine learning, we were able to estimate sensor orientation with error levels comparable to previous studies. This was achieved without the need for pre-calibration poses or additional sensor information. Moreover, we substantially reduced the inherent drift error typically associated with IMU-based trajectory estimation, through the application of upper limb swing kinematics within a 3-dimensional swing plane. Our research thus introduces a novel signal processing method for tracking rapid, wide-ranging motions, such as a golf swing, while maintaining user convenience. Our results could impact the burgeoning field of daily motion monitoring for health care, especially with the increasing prevalence of wearable devices like smartwatches.

## Data Availability

The datasets generated during and/or analyzed during the current study are not publicly available due to the reason that they involve handling experimental data with human subjects, but are available from the corresponding author on reasonable request.
