# Author Correction: Enhancing accuracy and convenience of golf swing tracking with a wrist-worn single inertial sensor

**DOI:** 10.1038/s41598-024-62799-1

**Published:** 2024-05-28

**Authors:** Myeongsub Kim, Sukyung Park

**Affiliations:** Department of Mechanical Engineering, Korea Advanced Institute of Science and Technology, Daejeon, 34141 South Korea

Correction to: *Scientific Reports* 10.1038/s41598-024-59949-w, published online 22 April 2024

The Acknowledgment section in the original version of this Article was omitted. The Acknowledgment section now reads:

This research is supported by the BK21 FOUR Program of the National Research Foundation Korea (NRF) grant funded by the Ministry of Education (MOE).

The original Article has been corrected.

